# TLR4 signaling modulates extracellular matrix production in the lamina cribrosa

**DOI:** 10.3389/fopht.2022.968381

**Published:** 2022-08-19

**Authors:** Emma K. Geiduschek, Paige D. Milne, Philip Mzyk, Timur A. Mavlyutov, Colleen M. McDowell

**Affiliations:** Department of Ophthalmology and Visual Sciences, University of Wisconsin-Madison, Madison, WI, United States

**Keywords:** ONH, TLR4 – toll-like receptor 4, TGFβ2, lamina cribrosa, ECM – extracellular matrix

## Abstract

The optic nerve head (ONH) is a place of vulnerability during glaucoma progression due to increased intraocular pressure damaging the retinal ganglion cell axons. The molecular signaling pathways involved in generating glaucomatous ONH damage has not been fully elucidated. There is a great deal of evidence that pro-fibrotic TGFβ2 signaling is involved in modulating the ECM environment within the lamina cribrosa (LC) region of the ONH. Here we investigated the role of signaling crosstalk between the TGFβ2 pathway and the toll-like receptor 4 (TLR4) pathway within the LC. ECM deposition was examined between healthy and glaucomatous human ONH sections, finding increases in fibronectin and fibronectin extra domain A (FN-EDA) an isoform of fibronectin known to be a damage associated molecular pattern (DAMP) that can activate TLR4 signaling. In human LC cell cultures derived from healthy donor eyes, inhibition of TLR4 signaling blocked TGFβ2 induced FN and FN-EDA expression. Activation of TLR4 by cellular FN (cFN) containing the EDA isoform increased both total FN production and Collagen-1 production and this effect was dependent on TLR4 signaling. These studies identify TGFβ2-TLR4 signaling crosstalk in LC cells of the ONH as a novel pathway regulating ECM and DAMP production.

## Introduction

Cupping of the optic nerve head (ONH), thinning and loss of the retinal nerve fiber layer, and characteristic visual field defects are all clinical features of glaucoma (1). Elevated intraocular pressure (IOP) is the most important risk factor for both the development and progression of glaucoma (2). Current glaucoma therapy involves decreasing IOP by suppression of aqueous humor formation, enhancing uveoscleral outflow or, most recently, directly targeting the trabecular meshwork. However, these therapies are not uniformly effective, and this therapy generally only slows the progression of vision loss over time (3–5). This highlights the crucial need for more effecting glaucoma treatments and an increased understanding of the molecular and cellular mechanisms of disease progression.

The ONH contains retinal ganglion cell (RGC) axon bundles and support tissues and cells. The lamina cribrosa (LC) is the main structural component of the ONH. The LC is a mesh-like connective tissue structure through which the RGC axons pass as they exit the eye to form the myelinated extraocular optic nerve. The LC is composed of a series of interconnected lamellar beams made up of elastin, collagens, laminin, and heparin sulfate proteoglycan. The LC provides support for the RGC axons and resident cells. The LC region is populated by three major cell types, glial fibrillary acidic protein (GFAP)-positive ONH astrocytes, microglia and α-smooth muscle actin (α-SMA) positive LC cells. The resident LC cells are located within the LC beams, and the ONH astrocytes are located both in longitudinal columns along RGC axon bundles and in transverse orientation investing multiple LC beams (6). Resident microglia are regularly spaced throughout the normal ONH in the walls of blood vessels, within the glial columns, and in the LC (7). The ONH region progressively remodels during glaucoma, leading to ONH cupping as well as mechanical failure and fibrosis of the LC; however, the cellular and molecular mechanisms responsible for this remodeling are not fully understood.

The ONH remains the most vulnerable point for the RGC axons, where they are most susceptible to elevated IOP, as the RGC axons have to turn 90° to enter the ONH and traverse the LC (8). This region is particularly susceptible to pressure because at the ONH, the sclera thins to form the ECM structure that allows RGC axons to exit the eye. Dysregulation of ECM in the LC causes increased fibrosis, elastosis, thickening of the connective tissue septae surrounding the ON fibers, and a thickening of the basement membranes involving altered collagen fibers and disorganized distribution and deposition of elastin, causing mechanical failure which in turn exacerbates ONH and RGC axon damage (6, 9, 10). Deposition of ECM causes the LC to initially undergo thickening and posterior migration. Eventual shearing and collapse of the LC plates leads to a thin fibrotic connective tissue structure/scar. ECM remodeling adversely affects the capacity of the LC to support RGC axons and predisposes RGCs to axonal compression, disruption of axoplasmic flow, and apoptosis.

Both the LC cells and ONH astrocytes are responsible for supporting the RGC axons by synthesizing growth factors and ECM (6, 11–13). Several cytokines are known to regulate the production and modulation of ECM, including Toll like receptor 4 (TLR4) signaling as previously described in other fibrotic diseases (14–16). TLR4 was first discovered as the receptor for lipopolysaccharide (LPS) (17), but can also be activated by endogenous ligands, known as DAMPs, damage associated molecular patterns. DAMPs are generated *in situ* as a result of injury, cell damage, ECM remodeling, and oxidative stress (18, 19). TLR4 is known to be expressed in astrocytes, microglia, and LC cells in the human ONH (20–22). TLR4 pathway related genes, downstream ECM genes, and DAMPs such as tenascin-C and heat shock proteins have been identified as differentially expressed in the human ONH and retina in glaucoma (23–25). Importantly, *TLR4* gene polymorphisms have been associated with enhanced glaucoma risk in some populations (26–28).

The role of TLR4 signaling in modifying the ECM and fibrotic environment has been studied in hepatic and renal fibrosis, scleroderma, as well as in *Tlr4* mutant mice (14–16). Recently, we identified TLR4 signaling as an important regulator of the ECM in the TM and ocular hypertension (29). In addition, DAMPs (including tenascin C, FN-EDA, heat shock proteins, and hyaluronan) have been shown to activate TLRβ and augment TGFβ signaling and downstream fibrotic responses (14, 30, 31), and DAMPs have been identified in the glaucomatous ONH of both mice and humans (32). Importantly, we previously identified the DAMP FN-EDA increases IOP in mice through the TLR4 signaling pathway (33). In addition, numerous studies have identified elevated aqueous humor levels of TGFβ2 in glaucoma patients (34–37). We and others have shown that TGFβ2 treatment of trabecular meshwork (TM) cells alters the ECM composition (29, 38–40) and induces ECM cross-linking (41–43). In the posterior segment, TGFβ2 is also the predominant TGFβ isoform in the ONH and astrocytes, LC cells, and activated microglia are known to express and secrete TGFβ2, with increased expression of TGFβ2 documented in the glaucomatous ONH (25, 44–47). TGFβ2 treatment of ONH astrocytes and LC cells *in vitro* increases ECM protein synthesis and secretion *via* canonical Smad signaling (44, 45). This dysregulation of ECM components could contribute to the ONH fibrotic environment in the LC in glaucoma. Here we demonstrate crosstalk between the TGFβ2 and TLR4 signaling pathways in primary ONH LC cells and show the DAMP, FN-EDA, is increased in the human glaucomatous LC suggesting this DAMP may have important implications in TLR4 activation and signaling in the glaucomatous ONH.

## Materials and methods

### Human donor eyes

Human donor eyes were obtained from the Lions Eye Bank of Wisconsin (Madison, WI) within 24 h of death. The eyes were obtained and managed in compliance with the Declaration of Helsinki. The human eyes used for IHC experiments ranged from 59 to 80 years old. Within 24 hours post time of death, human eyes are fixed in 4% paraformaldehyde for 24 hours at 4°C, rinsed with 1X PBS, then cryoprotected in 30% sucrose in PBS for another 48 hours at 4°C. The eye was embedded into optimum cutting temperature embedding medium (Sakura Finetek 4583, Sakura Finetek USA, Inc., Torrance, CA) in 25mm × 20mm × 5mm Tissue-Tek cryomolds (Sakura Finetek 4557) and frozen on a prechilled metal block. After which, cryosections at 10μm intervals were cut from the frozen eyecups before being stored at −80°C until immunostaining. Glaucoma diagnosis is based off patient medial history report and visual field defects. Primary human ONH LC cell strains were isolated from normal (nonglaucomatous) donor eyes (ages 59–74) from the Lions Eye Bank of Wisconsin or received as a kind gift from Dr. Abe Clark at the University of North Texas Health Science Center and characterized as previously described (11, 12, 48). All donor tissues were obtained and managed according to the guidelines in the Declaration of Helsinki for research involving human tissue. Cells were cultured and maintained in Ham’s F-10 growth media containing 10% FBS L-glutamine (0.292 mg/mL), and penicillin (100units/mL)/streptomycin (0.1mg/mL) in a humid chamber at 37°C in 5% CO_2_. The medium what replaced every 2–3 days.

### Immunohistochemistry

Standard procedures for IHC were utilized as previously described (33). The OCT was removed *via* two washes in ddH_2_O for 2 minutes each before dried by subsequent washing in 70% ethanol for 2 minutes, 100% ethanol for 2 minutes, and then left to dry at RT for 10 minutes. Sections were then rinsed with 1X PBS for 5 minutes before incubated in 0.1% Triton X (Sigma-Aldrich REFX100) at RT for 15 minutes to permeabilize cell membranes. Slides were then blocked in Superblock Blocking Buffer in PBS (REF37580, Thermo Fisher Scientific) for 60 minutes at RT before incubated at 4°C overnight with FN (Sigma-Aldrich Corp., F3648) at 1:250, and FN-EDA (Abcam, ab6328) at 1:100 dilution. Primary antibody was washed off with four rinses in 1X PBS for 5 minutes each before slides were incubated in the appropriate secondary antibody for 2 hours at RT; Alexa Fluor 488 donkey anti-rabbit IgG (A21206, Invitrogen – Thermo Fisher Scientific) at 1:200 dilution and Alexa Fluor 594 donkey anti-mouse IgG (A21203, Invitrogen – Thermo Fisher Scientific) at 1:200 dilution. Slides were washed 5 times in 1X PBS for 5 minutes each and mounted with Prolong Gold mounting medium containing DAPI (Invitrogen-Molecular Probes). Image acquisition was performed using Zeiss Axio Imager Z2 microscope. All images were taken at 40X magnification, scale bar represents 20μm.

### TLR4 inhibition and activation

Primary human ONH LC cells were grown to confluency and pretreated with the selective TLR4 inhibitor, TAK-242 (*In vivo*Gen, San Diego, CA, USA) at 15mM for 2 hours. TAK-242 is a cyclohexene derivative that specifically inhibits TLR4 signaling by binding to the intracellular domain of TLR4 and blocking downstream signaling. We previously performed a dose response curve using TAK-242 in primary human trabecular meshwork cells in culture and determined 15μM had the greatest inhibitory effect without affecting cell viability (29). Cells were then treated with TGFβ2 (5ng/mL) and/or TAK-242 (15μM) for 72 hours in serum-free medium. For TLR4 activation studies, hONH LC cultures were grown to confluency and treated with cellular fibronectin (cFN) (10μg/mL) containing the FN-EDA isoform (F2518; Sigma-Aldrich Corp., St. Louis, MO, USA) and/or TGFβ2 (5ng/mL), and/or TAK-242 (15μM) for 72 hours in serum-free medium. Western blot and immunocytochemistry experiments were performed as described below.

### Immunocytochemistry

Primary human ONH LC cells were seeded on 12-well plates on coverslips and grown to confluency. After undergoing TLR4 inhibition and activation treatments as previously described for 72 hours, cells were washed with 1X PBS, fixed with 4% paraformaldehyde (PFA), permeabilized with 0.95% Triton X-100 in PBS, and blocked using Superblock Blocking Buffer in PBS (REF37580, Thermo Fisher Scientific) for 60 minutes at room temperature. Cells were labeled overnight at 4°C with rabbit anti-Fibronectin (F3648, Sigma-Aldrich) at a 1:100 dilution, or rabbit anti-collagen-1 (NB600–408, Novus Biologicals) at a 1:100 dilution in Superblock Blocking Buffer in PBS. Treatment without the primary antibody was used as a negative control. Coverslips were then incubated for at room temperature 2 hours using Alexa Fluor 488 donkey anti-rabbit IgG (REFA21206, Invitrogen – Thermo Fisher Scientific) at a 1:200 dilution. Coverslips were mounted to slides with Prolong Gold mounting medium containing DAPI (Invitrogen-Molecular Probes). Image acquisition was performed using Zeiss Axio Imager Z2 microscope. Images were taken at X20 or X40 magnification; scale bar represents 20μm.

### Western blot analysis

Primary human ONH LC cells were treated as previously described above for 48 or 72 hours. Cell lysates were extracted using lysis buffer (Pierce RIPA Buffer, REF89901, Thermo Fisher Scientific; cOmplete Mini, EDTA-Free protease inhibitor cocktail, REF11836170001, Sigma-Aldrich; PhosSTOP REF04906837001 Sigma-Aldrich), and the Pierce^™^ BCA Protein Assay Kit (Thermo Fisher Scientific REF23225) was used to estimate protein concentrations of each sample. Each loading sample contained 10μg of protein and the appropriate amount of 4X Protein Loading Buffer (Li-Core REF928-40004). Samples were boiled for 10 minutes, then separated using a 4–12% Bolt^™^ Bis-Tris Plus mini gel (Invitrogen, NW04120BOX). Proteins from electrophoresed gels were transferred to polyvinylidene (PVDF) membranes (Bio-Rad Immun-Blot, REF1620177) for an hour at a constant 20V using the Mini Gel Tank wet transfer (Invitrogen, REFA25977). Membranes were left to dry for 45 minutes before using Revert^™^ 700 Total Protein Stain (Li-Core REF926-11021) to confirm equal loading for samples. After destaining of total protein stain, membranes were blocked for one hour at room temperature with Intercept Blocking Buffer (Li-Cor, REF927-60001). Membranes were immunolabeled overnight at 4°C with primary antibodies: GAPDH (1:5000, Cell Signal REF97166S), Fibronectin (1:1000, Sigma -Aldrich F3648), and/or FN-EDA (1:500, Abcam, ab6328), diluted in Intercept Blocking Buffer. Blots were washed three times for 5 minutes each with 1X TBS-T and then incubated for 1 hour with the appropriate secondary antibodies at a 1:20,000 dilution in Intercept Blocking Buffer (Li-Core Goat anti-rabbit IRDye 800CW; Li-Core Goat anti-rabbit IRDye 680RD; Li-Core Goat anti-mouse IRDye 800CW). Membranes were then imaged on a Licor OdysseyCLx system. Each experiment was repeated 2–3 times in each individual hONH LC strain, and a total of 2–3 independent hONH strains were tested. Band intensity for proteins of interest and total protein were measured using Image Studio Lite (LI-COR Biosciences, Lincoln, NE, USA). Each target protein densitometry value was normalized against either its corresponding GAPDH or total protein value as indicated, and the fold change was calculated to control. Fold changes are represented as the mean +/− SEM. Statistical significance determined by a 1-way ANOVA and subsequent Tukey’s *post hoc* analysis comparing all treatments.

## Results

### FN and the DAMP FN-EDA are increased in the LC region of the glaucomatous human ONH

**FIGURE 1:**
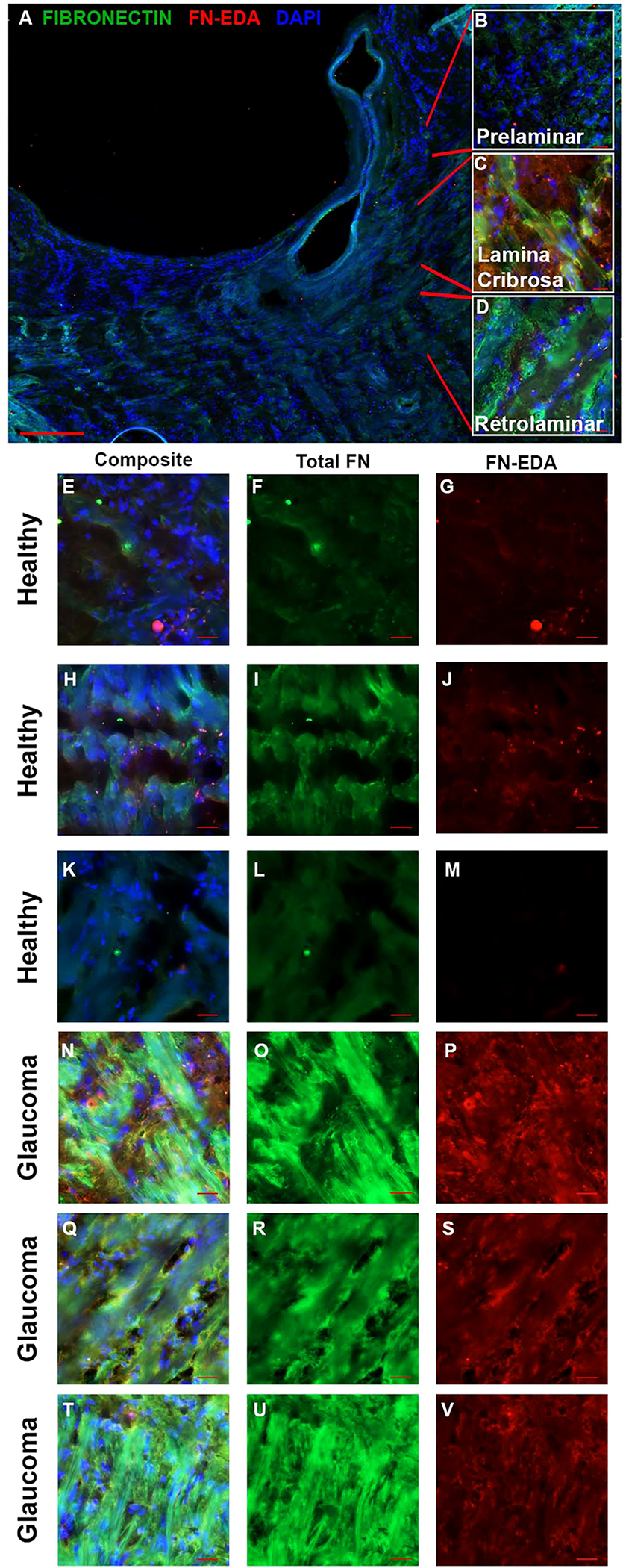
FN and FN-EDA expression in the LC region of normal and glaucomatous human ONH. **(A)** A cross-section of a hONH with **(B–D)** inserts of representative imaging locations of the **(B)** prelaminar, **(C)** LC, and **(D)** retrolaminar sections of the hONH. Immunohistochemistry images of the LC region of **(E–M)** healthy or **(N–V)** glaucomatous hONHs from donor eyes. Images show an increase in FN **(O, R, U)** and FN-EDA **(P, S, V)** expression in the LC region of glaucomatous eyes compared to healthy individuals (**F, I, L, G, J, M** respectively). *Scale bar* represents 20μm unless otherwise stated. [**(A)** 5X magnification, **(B–U)** 40X magnification. FN=green; FN-EDA=red; DAPI=blue.].

Increased fibrosis of the human ONH during glaucoma disease progression has been well established. The lamina cribrosa forms sieve-like layers of ECM which allow RGC axons to exit. The ECM is primarily made of collagens, elastin, and laminin (49), all of which have been shown to be increased in the glaucomatous ONH (50, 51). In addition, it is known that fibronectin is present in the LC region (52). In the present study, the human ONH (Figure 1A) including the prelaminar (Figure 1B), lamina cribrosa (Figure 1C), and retrolaminar (Figure 1D) regions were analyzed for changes in FN and FN-EDA expression. Composite images (Figures 1E, H, K, N, Q, T) show the overlay of FN and FN-EDA in the LC region. Here, we show an increase of fibronectin protein expression in the LC region of human glaucomatous donor eyes (Figures 1O, R, U) compared to normal non-glaucoma control donor eyes (Figures 1F, I, L). We also demonstrate an increase in the FN-EDA isoform, a known DAMP and activator of TLR4, in the LC region of human glaucomatous donor eyes (Figures 1P, S, V) compared to normal non-glaucoma control donor eyes (Figures 1G, J, M). These data suggest that FN and the FN-EDA isoform may have important implications in the development of glaucomatous ONH damage.

### Dissection and isolation of the human ONH generates monocultures of LC cells

**FIGURE 2:**
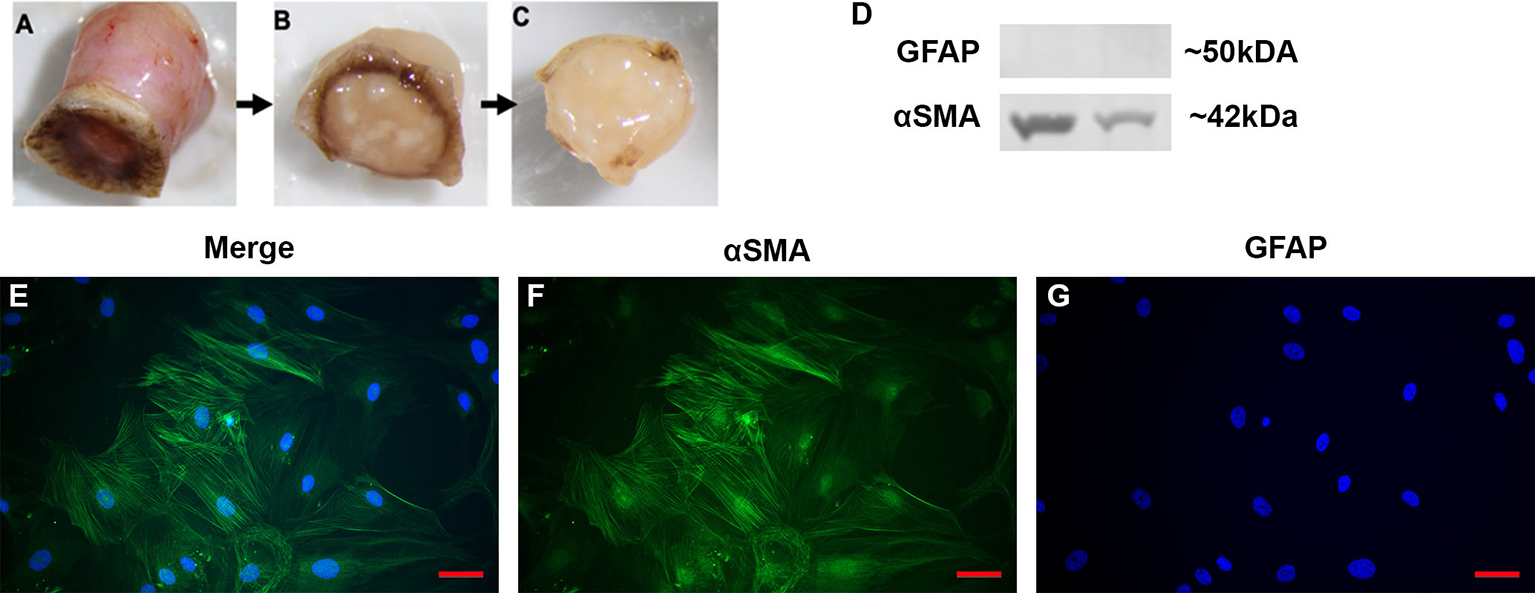
Isolation and characterization of LC cells from hONH explants. **(A–C)** Progressive removal of the RPE, peripapillary sclera, and ON from the ONH in initial isolation. The ONH explant was then cultured to isolate the LC section as previously described (48). **(D)** Representative western immunoblot from two different cell strains for GFAP and αSMA. ONH LC cells were positive for αSMA and negative for GFAP. **(E–G)** Immunocytochemistry staining of hONH LC cells were positive for αSMA **(F)** and negative for GFAP **(G)**. 40X magnification, *Scale bar* represents 100μm.

In order to test the function and role of DAMPs such as FN-EDA in LC cells, primary LC cells were isolated and cultured from human donor eyes. Following previously established protocols (48), the ONH was dissected and the ONH explant placed into culture to propagate LC cells (Figures 2A–C). The isolation and characterization of the monocultures of LC cells was performed as previously described by Lopez et al. (48). Here representative images (Figures 2E–G) and western blots (Figure 2D) show isolated LC cells are negative for GFAP and positive for αSMA, previously determined indicators of LC cells (48). In total we characterized 4 independent LC cell strains from different donors with no history of ocular disease.

### Inhibition of TLR4 signaling blocks TGFβ2-induced increases of ECM production in primary human ONH LC cells

**FIGURE 3:**
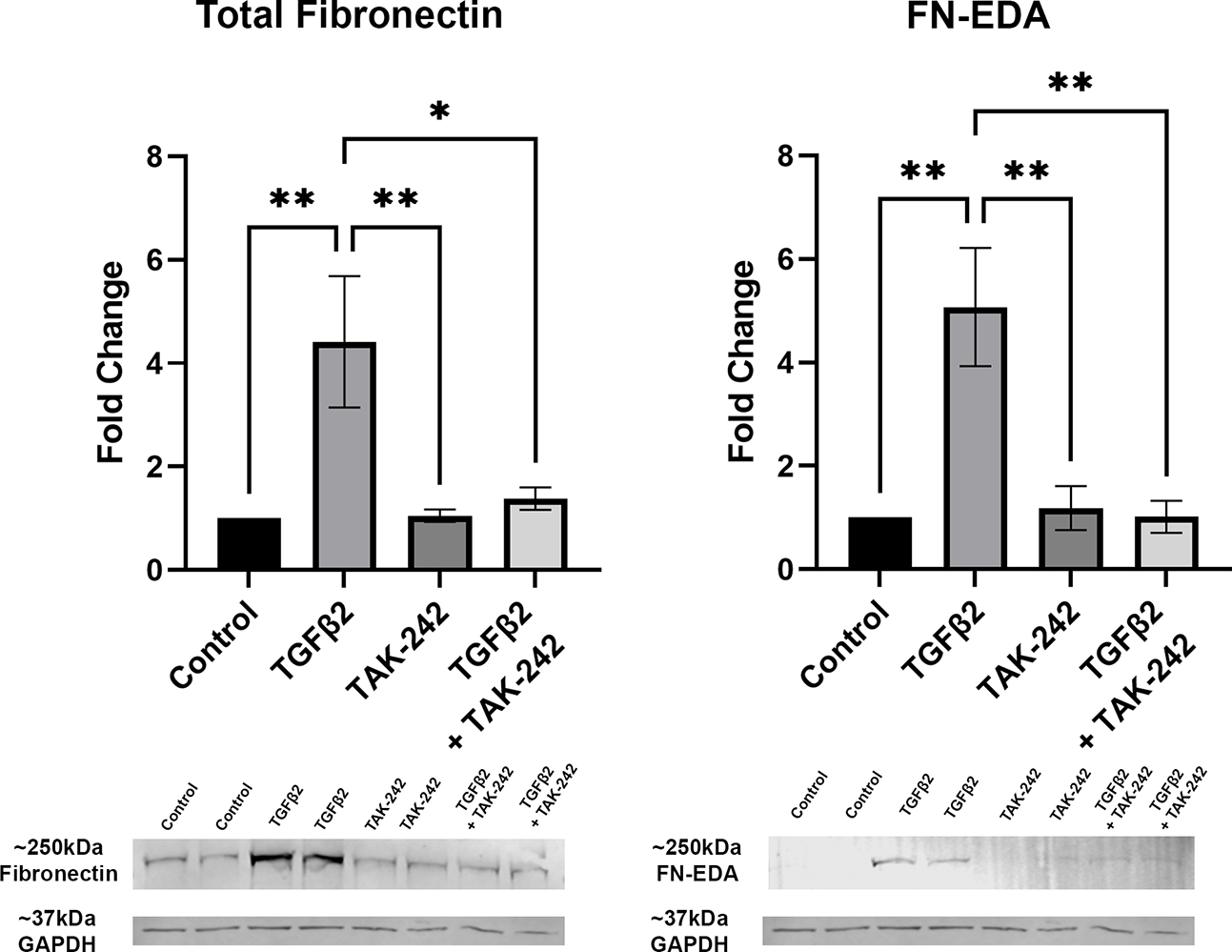
Inhibition of TLR4 blocks TGFβ2-induced ECM protein expression. **(A, B)** Primary hONH LC cells (*n* = 3 strains, each repeated in 2–3 independent experiments) were pretreated with TAK-242 for 2 hours, and subsequently treated with TGFβ2 (5 ng/mL) and/or TAK-242 (15μM) for 72 hours. Western immunoblot for **(A)** FN and **(B)** FN-EDA show that inhibition of TLR4 signaling *via* TAK-242 blocks the fibrotic effects of TGFβ2. Protein expression is normalized to GAPDH signal. **(C)** Representative immunoblots of FN and respective GAPDH protein expression, and **(D)** FN-EDA and respective GAPDH protein expression. Statistical significance was determined by 1-way ANOVA and Tukey’s *post hoc* analysis. *P < 0.05, **P < 0.01.

It is well established that TGFβ2 signaling increases in glaucoma and is known to affect the ONH during disease progression. Here, we show that inhibition of TLR4 by the selective inhibitor, TAK-242, blocks TGFβ2 dependent increases of total FN and the DAMP FN-EDA protein expression. Primary hONH LC cells were treated with TGFβ2 (5ng/mL) and/or TAK-242 (15μM) for 72 hours. As previously reported, TGFβ2 induces FN expression in LC cells (44, 45). However, TLR4 signaling inhibition significantly decreases FN (Figure 3A) and FN-EDA (Figure 3B) protein expression compared to GAPDH control (Figures 3C, D). Both FN and FN-EDA protein levels returned to control levels, with no significant differences between control and TAK-242 + TGFβ2 treated cells (*n* = 3 primary hONH LC cell strains, each repeated in 2–3 independent experiments). As expected, there was little FN-EDA in the control treated cells as the presence of FN-EDA is typically minimal in normal healthy cells (53). This data suggests that TLR4 signaling is necessary for TGFβ2 induced fibrosis in LC cells.

**FIGURE 4:**
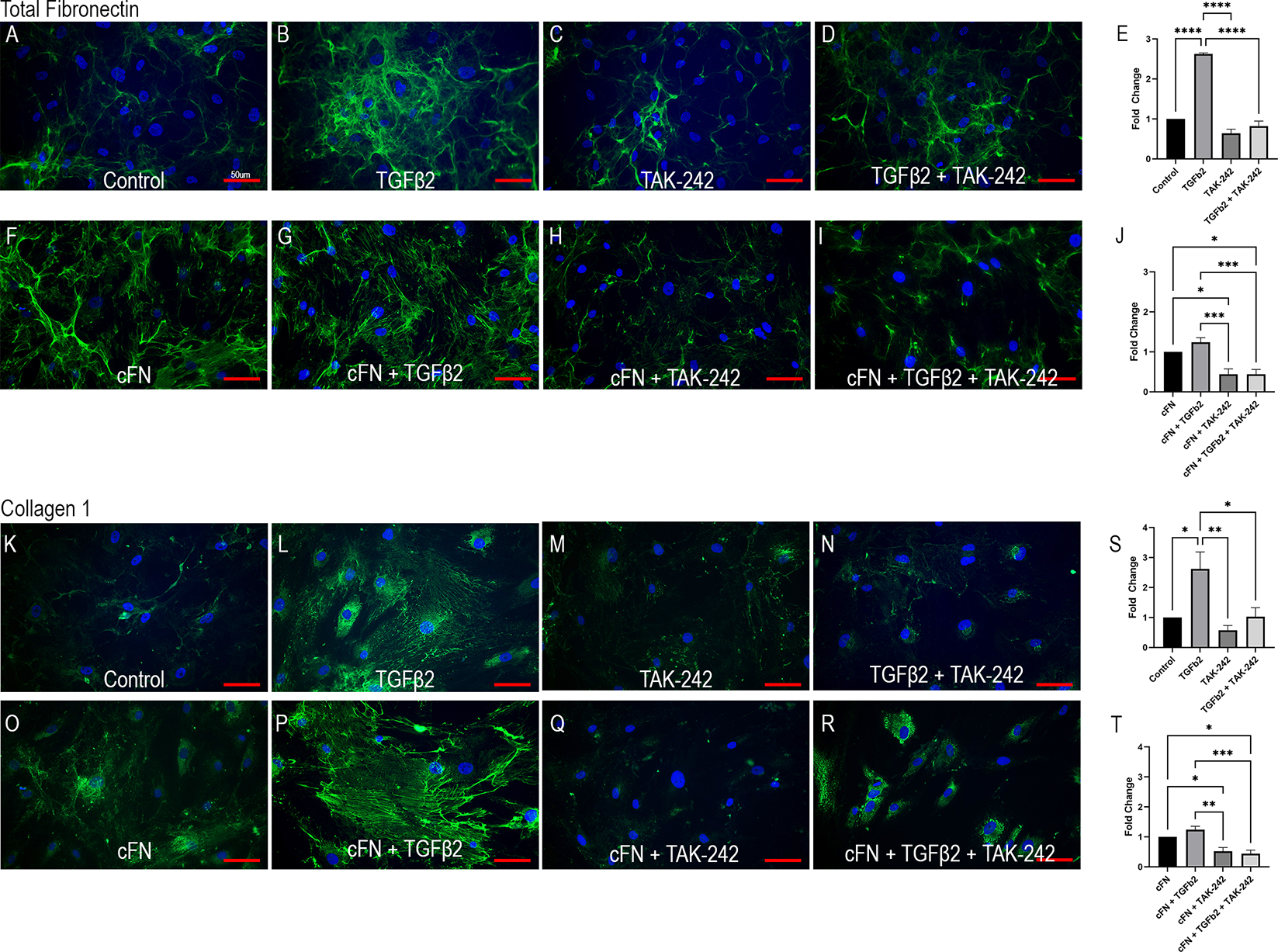
TLR4 signaling is necessary and sufficient for ECM production in hONH LC cells. Primary hONH LC cells (*n* = 4 cell strains) were grown to confluency on coverslips and left untreated for control **(A, K)** or treated with TGFβ2 **(B, D, G, I, L, N, P, R)** and/or cFN **(F–I, O–R)**. Additionally, cells were pretreated with the selective TLR4 inhibitor TAK-242 **(C, D, H, I, M, N, Q, R)** for 2 hours, followed by treatment with TGFβ2 and/or cFN for 72 hours. Quantification of the immunocytochemistry normalized to untreated control **(E, S)** and cFN treatment **(J, T)**. Scale bar represents 50μm, *P < 0.05, **P < 0.01, ***P < 0.001, ****P < 0.0001).

Cellular FN-EDA is an isoform of FN and has previously been shown to be a ligand for TLR4 (19, 54). Here we show that TLR4 inhibition prevents cFN induced ECM protein expression in primary ONH LC cells (Figure 4). Human ONH LC cells were grown to confluency on coverslips and treated with TGFβ2 (5ng/mL), TAK-242 (15μM), and/or cFN (10μg/mL). As expected, TGFβ2 increased FN (Figure 4B) and COL1 (Figure 4L) protein expression compared to control. This increase was dependent on TLR4 signaling, as the addition of the selective TLR4 inhibitor TAK-242 prevented TGFβ2-induced increases of both FN (Figure 4D) and COL1 (Figure 4N). The selective TLR4 inhibitor TAK-242 also prevented the cFN-induced increases of both FN and COL1 (Figures 4H, I, Q, R) compared to cFN treatment alone (Figures 4F, O). These expression changes were statistically significant (Figures 4E, J, S, T). Each experiment was repeated in 4 independent hONH LC cell strains. This data suggests a TGFβ2 – TLR4 signaling crosstalk in the ONH.

## Discussion

In primary open angle glaucoma (POAG) the extracellular matrix (ECM) of the LC is disturbed and remodeled resulting in mechanical failure and fibrosis. Cupping of the ONH and changes to the ECM of the LC are associated with disorganized and increased deposition of collagen and elastin fibers [4, 9, 10]. Early histological analysis of the glaucomatous ONH in humans and animal models demonstrated increases in collagen IV, elastin and tenascin (9, 25, 55, 56). In advanced glaucoma, histological analysis revealed a collapse of the LC plates and the formation of a fibrotic network of connective tissue. Both the LC cells and ONH astrocytes are responsible for supporting the RGC axons by synthesizing growth factors and ECM. Dysregulation of the ECM production and remodeling leads to glaucomatous changes in the ONH and RGC axon damage.

The pathogenic and molecular pathways responsible for the structural changes of the LC in POAG are not completely understood. However, it is well established that aqueous humor levels of TGFβ2 are elevated in POAG patients (34–37) and there is an increase in the levels of TGFβ2 in the ONH (25, 47). ONH astrocytes, LC cells, and activated microglia express and secrete TGFβ2 (44–46). Treatment of ONH astrocytes and LC cells with exogenous TGFβ2 increases ECM protein synthesis and secretion as well as phosphorylation of canonical Smad2/3 signaling proteins in both cell types (44, 45). Exogenous TGFβ2 also increases co-localization of pSmad2/3 with Co-Smad4 in the nucleus of ONH astrocytes and LC cells (44, 45). Knockdown of connective tissue growth factor (CTGF), a downstream signaling factor of TGFβ2, blocks the induction of ECM proteins by TGFβ2 in ONH astrocytes (57). The dysregulation of these ECM components could contribute to the fibrotic environment and basement membrane thickening in the LC of the ONH in glaucoma. In summary, these data suggest that TGFβ2 regulates the expression of ECM proteins in the ONH and the effects of TGFβ2 signaling are a major component in the development of glaucomatous ONH damage. Here we show that there is crosstalk between the TGFβ2 and TLR4 signaling pathways in ONH LC cells, and this signaling crosstalk may also extend to ONH astrocytes and microglia cells contributing to glaucomatous ONH damage.

**FIGURE 5:**
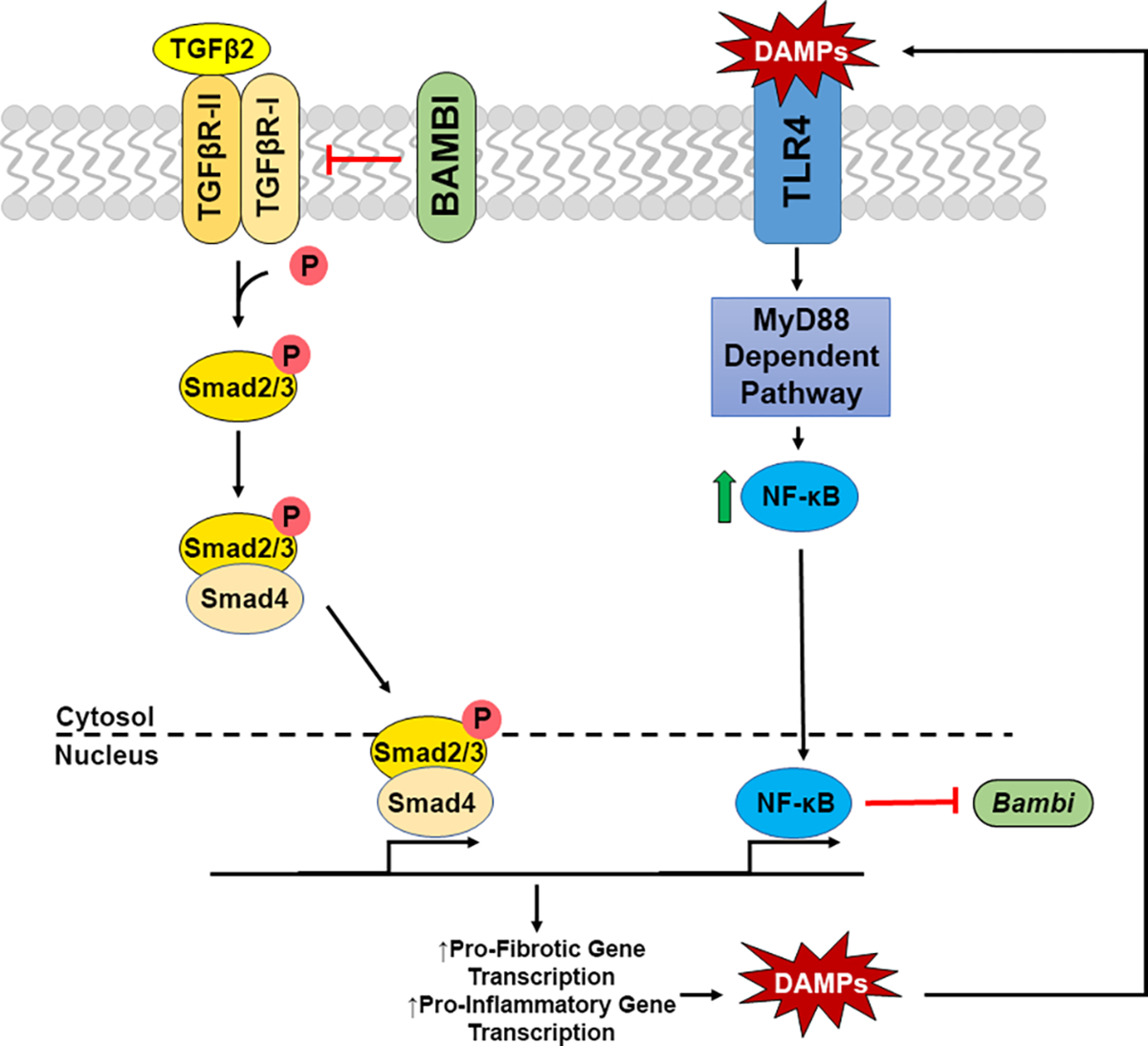
Crosstalk of TGFβ2 – TLR4 signaling in LC Cells. TGFβ2 activates the TGFβ receptor complex, phosphorylating Smad2/3. pSmad2/3 forms a complex with Smad 4, which translocates to the nucleus to act as a transcription factor increasing pro-fibrotic and pro-inflammatory gene transcription, including the production of DAMPs. These DAMPs are then able to activate TLR4 signaling, increasing NFκB through the MyD88 dependent pathway. NFκB translocates to the nucleus acting as a transcription factor, inhibiting the transcription of *Bambi*, which acts as a negative regulator of the TGFβ2 signaling cascade. Thus, these two pathways act in a feedforward loop.

TLR4 signaling is known to affect not only immune responses, but also initiate fibrotic responses in several disease states. In addition, certain alleles of the *TLR4* gene are associated with an increased risk of glaucoma in some populations (26–28). Interestingly, an increase in tenascin C, a large ECM glycoprotein and DAMP, has previously been reported to be increased in the glaucomatous ONH (25, 32). Here, we show an additional DAMP, FN-EDA, to be elevated in the glaucomatous ONH and modulate TLR4-TGFβ2 signaling crosstalk in LC cells. TLR4-TGFβ2 signaling crosstalk is likely regulated by the TGFβ pseudoreceptor BMP and activin membrane-bound inhibitor (BAMBI). *Bambi* is known to be expressed in both human ONH astrocytes and LC cells (58), and TLR4 activation downregulates Bambi expression, which enhances TGFβ signaling leading to increased ECM production (14, 15). BAMBI downregulation by TLR4 is regulated by a MyD88-NFκB-dependent pathway (15, 30, 31). BAMBI functions to inhibit TGFβ signaling by cooperating with SMAD7 and impairing SMAD3 activation, while knockdown of *Bambi* expression enhances TGFβ signaling (59). These data suggest a crosstalk between TLR4 and TGFβ signaling pathways in LC cells (Figure 5). Activation of TLR4 downregulates BAMBI leading to unopposed TGFβ signaling and fibrogenesis. Since the fibrotic response leads to the accumulation of endogenous TLR4 ligands such as FN-EDA and tenascin C, a feed-forward loop could develop leading to a further progression of the fibrotic response. Future studies will elucidate the exact molecular mechanism of TLR4 and TGFβ signaling crosstalk in the ONH.

The structure of the collagenous lamina cribrosa beams are disrupted in glaucoma. Increases of COL4 are seen in the LC region of ONH (56), and LC primary cell lines isolated from human glaucomatous optic nerve heads have significantly higher COL1 and COL5 mRNA expression than healthy control lines (60). COL1 protein expression also increases in other fibrotic diseases (61), and collagen VI staining also increases in other tissue types during stress (62). Concurrently, there is a marked loosening of the collagen matrix and significant loss of collagen fibers in the LC region of the ONH in POAG human tissue (55). Both COLVIII and COLXIII mRNA are downregulated when comparing LC cell mRNA synthesis from healthy versus glaucomatous derived primary cell cultures (60). This all suggests a disruption of collagen fibrils and density leading towards disease progression and pathogenic ECM modifications. Our results recapitulate previous literature in that TGFβ2 treatment increases COL1 deposition (22), and we further show that this increase is TLR4 dependent. In addition, we demonstrate that FN-EDA treatment is also sufficient in increasing COL1 expression in a TLR4-dependent manner. Future studies will look at other individual collagen subtype changes in glaucoma progression in the LC.

In conclusion, we show novel findings highlighting increased total FN and increased FN-EDA expression in the LC region of the glaucomatous human ONH. TGFβ2-TLR4 crosstalk in hONH LC cells is involved in the production and regulation of the ECM. Both TGFβ2 and cFN containing the EDA isoform can increase ECM protein expression in LC cells, as well as increase the production of the DAMP FN-EDA, and inhibition of TLR4 blocks these effects. These results provide insights into a novel pathway in the ONH region that could be driving glaucoma disease progression and eventual loss of vision. Understanding these cellular signaling mechanisms behind ONH damage offer new targets for developing further treatment therapies.

## Data Availability

The original contributions presented in the study are included in the article/supplementary material. Further inquiries can be directed to the corresponding author.
